# TaPR1 Interacts With TaTLP1 *via* the αIV Helix to Be Involved in Wheat Defense to *Puccinia triticina* Through the CAPE1 Motif

**DOI:** 10.3389/fpls.2022.874654

**Published:** 2022-05-26

**Authors:** Fei Wang, Songsong Shen, Cunpeng Zhao, Zhongchi Cui, Linshuo Meng, Wenyue Wu, Daqun Liu, Haiyan Wang

**Affiliations:** ^1^College of Plant Protection, Technological Innovation Center for Biological Control of Crop Diseases and Insect Pests of Hebei Province, Hebei Agricultural University, Baoding, China; ^2^Institute of Cotton, Hebei Academy of Agriculture and Forestry Sciences, Shijiazhuang, China

**Keywords:** TaPR1-4, αIV helix, CAPE1, resistance mechanisms, *Triticum aestivum*, *Puccinia triticina*

## Abstract

Pathogenesis-related (PR) proteins play important roles in plant defense response and systemic acquired resistance (SAR). PR1 has antifungal activity against many plant pathogens. In our previous study, RNA sequencing (RNA-seq) was conducted on resistant wheat line TcLr19 and sensitive wheat cultivar Chinese Spring inoculated with *Puccinia triticina* (*Pt*) race PHNT. In this study, seven salicylic acid (SA)-induced *TaPR1* genes involved in plant disease resistance were found in the RNA-seq library. Quantitative PCR (qPCR) results showed that *TaPR1-4* was most induced by *Pt* among these seven *TaPR1* genes in the incompatible interaction. Yeast two-hybrid (Y2H) results showed that TaPR1-4 interacted with TaTLP1 *via* the αIV helix. Protein-mediated phenotyping assays *in vivo* and antifungal activity *in vitro* demonstrated that wheat leaves infiltrated with pure TaPR1-4 protein developed significantly less disease compared to control leaves. This effect was correlated with a strong increase in defense gene expression, and resistance activity was dependent on the CAPE1 motif located in the C-terminal region of TaPR1-4. These findings increase current knowledge regarding the interaction of TaPR1 and TaTLP1 and provide new insights on the role of TaPR1 protein in the resistance of wheat to *Pt*.

## Introduction

Pathogenesis-related proteins (PRs) are a series of proteins that accumulate in plants under biotic or abiotic stresses. They participate in plant disease resistance by solidifying cell walls, enhancing antifungal activity, or participating in cell signal transduction. They are among the most important components of the plant defense response system (Christensen et al., 2002). So far, 17 gene families designated as *PR* genes have been identified from plant species (Roggero and Pennazio, 1989; Hakim et al., 2018). *PR1*, *PR2*, and *PR5* are used as marker genes of systemic acquired resistance (SAR) and are upregulated in response to various pathogens.

PR1 is a member of the cysteine-rich secretory proteins, antigen 5, and the PR1 (CAP) superfamily (Chien et al., 2015). CAP-derived peptide 1 (CAPE1) not only triggers antiherbivore responses but also primes antipathogen activity to prevent infection at wound sites (Chen et al., 2020). In hexaploid wheat (*Triticum aestivum* L.), 23 *PR1*-like (*TaPR1*) genes are further classified into three major groups, and *TaPR1-1*–*TaPR1-5* have disease resistance functions in wheat (Lu et al., 2011). *TaPR1* gene transcripts rapidly accumulate to high levels in response to biotic or abiotic stress and confer antifungal activity in various plant species (Wang J. et al., 2019; Ghorbel et al., 2021). Esmail et al. (2020) reported that *PR1* expression was upregulated in resistant wheat cultivars at the seedling and adult stages following stripe rust infection. In addition to responding to biotic and abiotic stresses, PR1 protein also plays a role in plant growth and development. Expression levels of *TaPR1* in the wheat line TcLr35 increased from the 1-leaf to tillering stage, then significantly increased at the booting stage, and peaked at the mature stage (Li et al., 2016).

The expression of thaumatin-like proteins (TLPs) belonging to the PR5 family can also be induced in response to biotic and abiotic stresses (Hakim et al., 2018). Transcription of *TaPR5* wheat cultivar Suwon 11 was upregulated in incompatible interactions and also induced by abiotic stress such as phytohormones and stress stimuli (Wang et al., 2010). Muoki et al. (2021) reported that heterologous expression of *CsTLP* (*Camellia sinensis*) improved seed yield under drought stress in transgenic lines of *Arabidopsis*. Accumulation of tomato TLPs (PR-NP24) induced by salt treatment promotes resistance to fungal pathogens (An et al., 2019). Zhang et al. (2019) reported that higher expression levels of *TLPs* and *PR1a* increased the resistance to spotted leaf, a disease-mimic condition in barley.

Leaf rust, caused by *Puccinia triticina* (*Pt*), is one of the most destructive diseases in wheat. Cultivating resistant cultivars is an effective way to control this disease, but race-specific resistance can be overcome quickly due to the rapid evolution of *Pt* populations. Understanding the molecular mechanisms of the interaction between wheat and *Pt* is critical to controlling this disease. In our previous study, *TaPR1-4* (Gene ID: HQ848391) and *TaTLP1* (Gene ID: KJ764822) were identified from the wheat near-isogenic line TcLr19 following infection with *Pt* race PHNT. Zhang et al. (2018) reported that *TaTLP1* participated in *Lr35*-mediated adult-plant resistance to *Pt* and that overexpression of *TaTLP1* enhanced resistance to *Pt* compared to a susceptible control (Cui et al., 2021). We earlier found that the interaction of the TaPR1 and TaTLP1 proteins contributed to resistance to *Pt* in a reactive oxygen species (ROS)-dependent manner (Wang F. et al., 2020). In this study, we aimed to dissect the interaction of TaPR1 and TaTLP1 and to identify the key functional region in TaPR1-4 that contributed to resistance. We believe our findings will provide new insights into the molecular mechanisms of *TaPR1-4* in resistance to *Pt* and will help to further characterize and exploit PR1-mediated defense signaling in protecting wheat against leaf rust.

## Materials and Methods

### Plant Materials and *Puccinia triticina* Race

Wheat near-isogenic line TcLr19 (Tc*6/RL6040), susceptible wheat line Chinese Spring, *TaTLP1*-overexpressing transgenic line (TaTLP1-OE), and wild type Jinan Wheat No. 1 (JW1) are preserved in the Laboratory of Wheat Leaf Rust, Hebei Agricultural University. *Pt* race PHNT (isolate 07-10-426-1) was used in all tests according to methods outlined by Roelfs and Martell (1984). The second seedling leaves inoculated with PHNT or distilled water (control) for RNA extraction were harvested at 0, 24, 48, 72, 96, and 120 h post-inoculation (hpi). All samples were immediately frozen in liquid nitrogen and stored at −80°C. Each treatment included three independent biological replicates.

### Data Analysis

RNA-seq was used to analyze 7 *TaPR1* genes involved in the *Lr19*-mediated resistance to *Pt*. The molecular size and isoelectric points (p*I*) of seven *TaPR1* genes were predicted using ProtParam.^1^ Subcellular localization and chromosome locations were determined using EnsemblPlants.^2^ The signal peptides were identified using SignalP 5.0.^3^

### RNA Isolation and Quantitative PCR

Total RNA was extracted using an M5 Plant RNeasy Complex Mini Kit (Mei5bio, Beijing) according to the manufacturer’s instructions. cDNA was synthesized using the M5 Super Plus qPCR RT kit with gDNA remover (Mei5bio, Beijing). qPCR was conducted using 2 × M5 HiPer Realtime PCR Super Mix (Mei5bio, Beijing) with an ABI QuantStudio 5 instrument (ABI, Waltham, MA, United States). Expressions of *TaPR1-1*, *TaPR1-4*, *TaPR1-7*, *TaPR1-9*, *TaPR1-16*, *TaPR1-19*, and *TaPR1-20* were investigated, and the wheat glyceraldehyde-3-phosphate dehydrogenase (*GAPDH*, Gene ID: AF251217) gene was used to calibrate the expression levels of queried genes, as previously described (Gao et al., 2015; Supplementary Table 1). Data were analyzed using the 2^–ΔΔCT^ method (Livak and Schmittgen, 2002). The statistical significance of differences was calculated using one-way analysis of variance (ANOVA) and Duncan’s multiple range test (DMRT) with *p* < 0.05 in SPSS 26.0 (IBM SPSS Statistics, IBM). For each treatment, three technical repeats and three independent biological replicates were used for analysis.

### Subcellular Localization of *TaPR1-4*

Green fluorescent protein (GFP) fusion constructs were produced by cloning the coding sequence of *TaPR1-4* into the vector pEarlyGate103 (with GFP-trap). Resuspended *Agrobacterium tumefaciens* GV3101 carrying pEarlyGate103-TaPR1-4 and pEarlyGate103 at a final OD_600_ = 0.8. *Nicotiana benthamiana* infiltrations were performed on plants that were 4–5 weeks old. pEarlyGate103 was used as a negative control. The fluorescence in leaves of *N*. *benthamiana* was monitored 48 h after agroinfiltration and then imaged directly using a confocal laser scanning microscope (Olympus FluoView FV1000).

### Yeast Two-Hybrid Assays

The Matchmaker Gold Yeast Two-Hybrid System (Clontech, Japan) was used to verify the interaction between TaTLP1 and different truncated TaPR1-4 mutants, which included C-terminal deletions of residues 113–164 (C_Δ__113–164_-TaPR1-4), 119–164 (C_Δ__119–164_-TaPR1-4), 128–164 (C_Δ__128–164_-TaPR1-4), and 143–164 (C_Δ__143–164_-TaPR1-4). These mutants were generated by PCR amplification and subcloned into pGBKT7 as bait (Supplementary Table 1). TaTLP1 was transformed into pGADT7 as prey. The bait and prey plasmids were co-transformed into yeast strain Y2HGold according to the manufacturer’s instructions. A series of site-specific mutant variants (A114S, A115R, G116A, K117A, and V118S) of TaPR1-4 were synthesized, ligated to the pGBKT7 vector, transformed into yeast cells, and assayed for growth on synthetic dropout SD/-Trp-Leu and SD/-Trp-Leu-His-Ade plates containing X-α-galactosidase (X-α-Gal) and aureobasidin A (AbA).

### Expression and Purification of TaPR1-4 and Mutant Proteins in *Escherichia coli*

According to the secondary structures of TaPR1-4 protein, truncated TaPR1-4 variants in the N- or C-terminal region and wild type TaPR1-4 were generated by PCR amplification and cloned into pGEX-6P-3 (with GST-tag) (Supplementary Table 1). The recombinant proteins were expressed in *E. coli*. Crude proteins were induced by isopropyl β-d-1-thiogalactopyranoside (IPTG) with a final concentration of 0.5 mmol L^–1^ (Salcedo et al., 2017). A GST-Agarose Label kit (TRAN, Beijing) was used to bind GST-tagged protein following the manufacturer’s instructions. The purified protein products were separated by 15% SDS-PAGE and visualized using Coomassie Blue staining.

### Antifungal Activity Assays *in vitro*

*Puccinia triticina* race PHNT was tested for disease response in the presence of TaPR1-4, N_Δ__25–64_-TaPR1-4, C_Δ__119–164_-TaPR1-4 (-αIV), C_Δ__128–164_-TaPR1-4 (+αIV), and CAPE1 peptide, respectively. According to the experiment used to detect germination of urediniospores, petri dishes were filled with 20 ml of agar medium containing purified TaPR1-4, N_Δ__25–64_-TaPR1-4, C_Δ__119–164_-TaPR1-4 (-αIV), C_Δ__128–164_-TaPR1-4 (+αIV), and CAPE1 peptide (1 mg/ml). The plates were incubated at 25°C in the dark, and the germination of spores and hyphal length were observed using the Nikon Ti2-LAPP Ti2 Laser Application System (Nikon Corporation, Minato-ku, Tokyo, Japan). Negative controls were also carried out using sterile water, elution buffer, and GST tag protein.

### Antifungal Activity Assays *in vivo*

To identify the antifungal activity of CAPE1 in TaPR1-4 *in vivo*, the susceptible wheat line Chinese Spring was used for infiltration assays. TaPR1-4, CAPE1, and C_Δ__128–164_-TaPR1-4 (+αIV) proteins were diluted to 0.1 mg/ml in 1 × PBS buffer (137 mM NaCl, 2.7 mM KCl, 10 mM Na_2_HPO_4_, and 2 mM KH_2_PO_4_) and infiltrated into the abaxial side of the first leaves of 14-day-old seedlings of Chinese Spring using a 0.5-ml syringe and GST proteins as a negative control. Inoculations with *Pt* race PHNT were carried out 24 h after protein infiltration, and disease responses were observed after 14 days. Leaves at 0, 24, 48, and 120 hpi were sampled and RNA extracted, then transcription levels of several resistance-related genes, including *TaPR1-4*, *TaTLP1*, and *TaSOD* (superoxide dismutase, SOD), were analyzed following protein infiltration and inoculation with *Pt*. A similar experiment was conducted on 21-day-old *TaTLP1*-OE and JW1 wheat lines to further explore the role of CAPE1 in the TaTLP1-mediated resistance response, then the transcription levels of *TaPR1-4*, *TaCAT* (catalase, CAT), *TaSOD*, and *TaNOX* (NADPH oxidase, NOX) at 0, 24, 48, and 120 hpi were investigated using qPCR. In all these experiments, each treatment included 3–5 plants and was repeated at least two times. The numbers of urediniospores were quantified using Image J software.

### Histological Observation of Fungal Growth

Harvested samples were decolorized as described previously (Wang et al., 2007). Hyphae were stained using Fluorescent Brightener 28. Autofluorescence of attacked mesophyll cells was observed in necrotic areas using an Olympus IX-53 microscope (Olympus Corporation, Tokyo, Japan) (excitation filter, 488 nm; dichromic mirror, 510 nm; and barrier filter, 520 nm). Necrotic areas and infection areas were quantified using Image J software. For each treatment, at least 50 different infection sites were examined on each of five randomly selected leaf segments.

### Detection of H_2_O_2_ Accumulation

To detect H_2_O_2_ accumulation, 3, 3-diaminobenzidine (DAB; Solarbio, Beijing) staining was conducted following the protocols described previously (Thordal Christensen et al., 1997) and was then viewed by differential interference contrast optics. Areas of H_2_O_2_ were quantified using Image J software. A minimum of 50 infection sites were examined on each of five randomly selected leaf segments for every treatment.

## Results

### The *TaPR1* Genes Involved in the *Lr19*-Mediated Resistance to *Puccinia triticina* by RNA-Seq

In our previous study, transcriptome sequencing was conducted on the resistant line TcLr19 and the sensitive variety Chinese Spring inoculated with *Pt* race PHNT (Raw sequence reads have been deposited in the NCBI Sequence Read Archive under the BioProject PRJNA694214). In an RNA-seq library, seven *TaPR1* genes, namely *TaPR1-1*, *TaPR1-4*, *TaPR1-7*, *TaPR1-9*, *TaPR1-16*, *TaPR1-19*, and *TaPR1-20*, were identified and divided into three groups following Lu et al. (2011) (Supplementary Table 2). Amino acid sequence analyses indicated that the open reading frames (ORFs) of these *TaPR1* genes ranged from 495 to 522 base pairs (bp), and molecular weights ranged from 17 to 18 kDa. Predicted isoelectric points (p*I*) of the proteins by Expasy ranged from 4.2 to 8.7 (Supplementary Table 2). Predicted subcellular localization by EnsemblPlants showed that all seven *TaPR1* proteins were located in the apoplastic space. Predicted chromosome locations in EnsemblPlants indicated that *TaPR1-1* and *TaPR1-7* were located in chromosome 5B, *TaPR1-9* and *TaPR1-19* were located in chromosome 5A, *TaPR1-16* was located in chromosome 5D, and *TaPR1-4* and *TaPR1-20* were located in chromosome 7D. Signal peptide (SP) structure analysis using SignalP 5.0 indicated that all TaPR1 proteins contained an SP motif at the N-terminus (Supplementary Table 2).

### Expression Profiles of the *TaPR1* Genes Induced by *Puccinia triticina*

**FIGURE 1:**
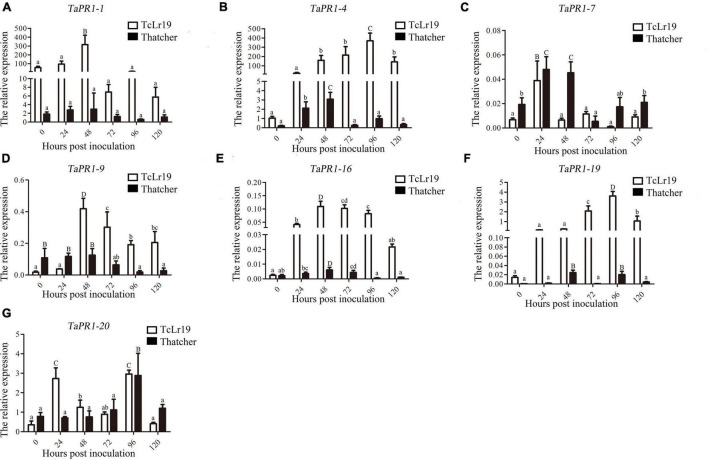
The expression profiles of seven *TaPR1* genes in incompatible and compatible interactions at different times post-inoculation. **(A–G)** Relative expression of TaPR1-1, TaPR1-4, TaPR1-7, TaPR1-9, TaPR1-16, TaPR1-19, and TaPR1-20 after inoculated with Pt is presented as fold change relative to mock-inoculated plants at 0 hpi. The y-axis indicates the amount of *TaPR1* genes transcript normalized to the *GAPDH* gene. The x-axis indicates sampling time. Values are means ± SEM of three independent biological replicates. Significant differences were assessed using one-way analysis of variance (ANOVA) and Duncan’s multiple range test (DMRT) with *p* < 0.05.

Transcription levels of the seven *TaPR1* genes in TcLr19 following infection with *Pt* were determined by qPCR. *TaPR1-1*, *TaPR1-9*, and *TaPR1-16* expression peaked at 48 hpi and then decreased in the incompatible combination. However, transcription of these genes in the Thatcher control, which is susceptible to all tested *Pt* races, was almost unchanged over the same time periods (Figures 1A,D,E). *TaPR1-7* and *TaPR1-20* were highly expressed in both TcLr19 and Thatcher (Figures 1C,G). *TaPR1-4* and *TaPR1-19* expression peaked at 96 hpi in TcLr19 when the expression level was 253.93-fold that at 0 hpi, and the expression of *TaPR1-4* at 96 hpi was 358.08-fold higher than at 0 hpi. However, the expression levels of *TaPR1-4* and *TaPR1-19* in Thatcher were lower than that in TcLr19 (Figures 1B,F). Our findings demonstrated that *TaPR1-4* is most induced by *Pt*. In accordance with our previous studies (Gao et al., 2015; Wang F. et al., 2020), *TaPR1-4* was used for further study.

### Localization of TaPR1-4 Protein in Plant Cell

To further assess the secretion and localization of TaPR1-4 protein *in vivo*, we generated the fusion construct pEarlyGate103-TaPR1-4 in which *TaPR1-4* was fused to GFP at its C-terminus for the *agrobacterium*-mediated transformation of *N*. *benthamiana*. The empty vector pEarlyGate103 was used as a negative control. Confocal microscopic observation showed that the control GFP protein was localized in both the cytoplasm and nucleus (Supplementary Figure 1). In contrast, pEarlyGate103-TaPR1-4 signals were clearly visualized in the apoplastic space (AP), as evidenced by the plasmolysis (Supplementary Figure 1), and no GFP signals were observed in the nucleus. Based on these observations, we concluded that *TaPR1-4* was secreted outside of plant cell, which was consistent with the predicted subcellular localization.

### The αIV Helix Is Indispensable in the Binding of TaPR1-4 and TaTLP1

**FIGURE 2:**
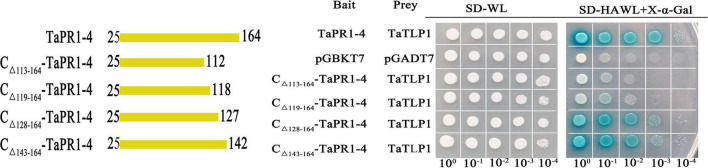
The αIV helix of TaPR1-4 is indispensable for the interaction with TaTLP1. Truncated TaPR1-4 constructs were generated, including C-terminal deletion of residues 113–164 (C_Δ__113–164_-TaPR1-4), deletion of residues 119–164 (C_Δ__119–164_-TaPR1-4), deletion of residues 128–164 (C_Δ__128–164_-TaPR1-4), and deletion of residues 143–164 (C_Δ__143–164_-TaPR1-4) as bait, with TaTLP1 as prey. Each of the constructs was co-transformed with TaTLP1 into yeast. TaPR1-4 (bait) and TaTLP1 (prey) are the positive controls; pGADT7 and pGBKT7 are used as the negative controls. All transformants can grow on synthetic dropout medium without leucine and tryptophan (SD-WL) medium. Yeast colonies that were able to grow on a selective medium (SD medium without leucine, tryptophan, histidine, and adenine supplemented with X-α-Gal and Aureobasidin A [SD-HAWL]) and displayed blue coloration confirmed the protein–protein interaction.

In a previous study, we showed that 15 amino acids from 113 to 127 located in the C-terminal region of TaPR1-4 are required for interaction with TaTLP1 (Wang F. et al., 2020). The detailed structure of TaPR1-4 was analyzed to further identify critical residues of TaPR1-4 interacting with TaTLP1. We found that amino acids A114–V118 were exposed on the surface (Supplementary Figure 2). Site-specific mutagenesis with a focus on residues A114–V118, respectively, was performed as these amino acids were less likely to be structurally disruptive (Protein ID: Q94F73). Yeast two-hybrid (Y2H) results showed that TaTLP1-pGADT7 and TaPR1-4-pGBKT7 as positive controls produced blue coloring (Figure 2, rows 1 and Supplementary Figure 3, rows 1), whereas interactions between all five mutants and TaTLP1 were weakened, but not negative (Supplementary Figure 3, rows 5–9). The negative controls pGADT7 and pGBKT7 caused a loss of blue coloration (Figure 2, row 2 and Supplementary Figure 3, row 2). These results indicate that a single residue from A114 to V118 cannot abolish the interaction between TaPR1-4 and TaTLP1.

Kirby et al. (2002) reported that the αIII and αIV helices in the PR1 fold likely interact with other proteins. Truncated variants C_Δ__119–164_-TaPR1-4 without the αIV helix structure (-αIV) and C_Δ__128–164_-TaPR1-4 including the αIV helix structure (+αIV) were generated to identify the effect of αIV helix on the interaction between TaPR1-4 and TaTLP1. Truncated variants C_Δ__113–164_-TaPR1-4 and C_Δ__143–164_-TaPR1-4 from our previous study (Wang F. et al., 2020) were used as controls (Supplementary Figure 4 and Figure 2). Y2H results showed that the truncated variants C_Δ__128–164_-TaPR1-4 (+αIV) and C_Δ__143–164_-TaPR1-4 interacted with TaTLP1 (Figure 2, rows 5–6), whereas C_Δ__113–164_-TaPR1-4 failed to interact with TaTLP1, and C_Δ__119–164_-TaPR1-4 (-αIV) with TaTLP1 still remained a trace of weak interaction (Figure 2, rows 3–4). These results showed that interaction between C_Δ__119–164_-TaPR1-4 (-αIV) and TaTLP1 was abolished or extremely weakened, indicating that the αIV helix in TaPR1-4 contains critical residues for binding with TaTLP1, which means the critical residue of TaPR1-4 binding with TaTLP1 was further narrowed based on our previous study.

### CAPE1 Is a Key Functional Region in TaPR1-4 Contributed to Resistance to *Puccinia triticina*

**FIGURE 3:**
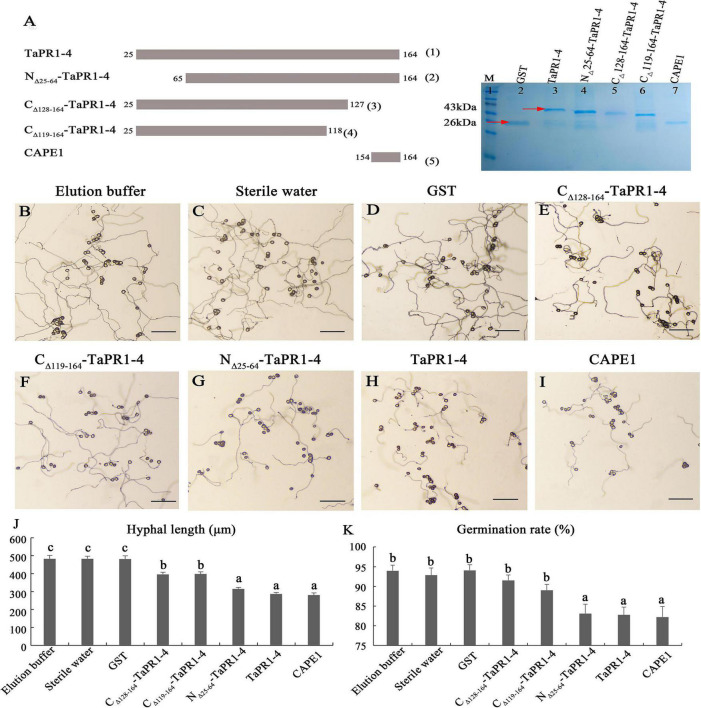
The antifungal activity of TaPR1-4 *in vitro*. **(A)** Schematic of constructing site-directed mutagenesis and SDS-PAGE electrophoresis of purified proteins. Truncated TaPR1-4 constructs were generated including N-terminal deletion of residues 25–64 (N_Δ__25–64_-TaPR1-4), C-terminal deletion of residues 119–164 (C_Δ__119–164_-TaPR1-4), and deletion of residues 128–164 (C_Δ__128–164_-TaPR1-4). Lane 1 to lane 7 show that Marker, GST, TaPR1-4, N_Δ__25–64_-TaPR1-4, C_Δ__128–164_-TaPR1-4, C_Δ__119–164_-TaPR1-4, and CAPE1. The proteins were stained with Coomassie Brilliant Blue. Red arrows mark protein size. **(B–I)** The antifungal activity of TaPR1-4 on the 2% agar medium under 25°C dark for 12 h treated with elution buffer, sterile water, GST, C_Δ__128–164_-TaPR1-4, C_Δ__119–164_-TaPR1-4, N_Δ__25–64_-TaPR1-4, TaPR1-4, and CAPE1, respectively. Photographs were taken at 10× magnification. Scale bar, 100 μm. **(J,K)** The hyphal length and urediniospores germination rate were counted using Excel. Values are means ± SEM of three independent biological replicates. Significant differences were assessed using one-way analysis of variance (ANOVA) and Duncan’s multiple range test (DMRT) with *p* < 0.05.

**FIGURE 4:**
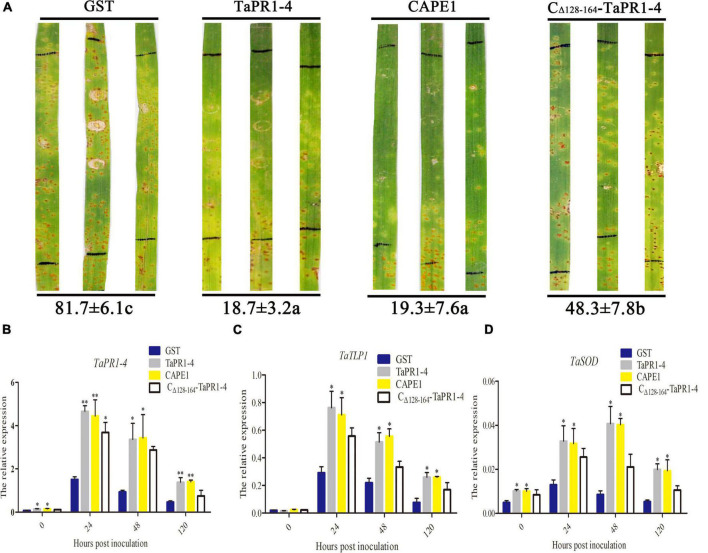
Antifungal activity of CAPE1 *in vivo*. **(A)** GST, TaPR1-4, CAPE1, and C_Δ__128–164_-TaPR1-4 (0.1 mg/ml) were infiltrated into the first leaves of Chinese Spring. Black lines indicate the infiltration zones. The numbers of urediniospores at 14 dpi were quantified using Image J software. **(B–D)** Expression fold change of *TaPR1-4*, *TaTLP1*, and *TaSOD* measured after GST, TaPR1-4, CAPE1, and C_Δ__128–164_-TaPR1-4 pure protein into susceptible wheat Chinese Spring and inoculated with *Pt*. Relative expression was expressed as fold change relative to mock-inoculated plants at 0 hpi. The y-axis indicates the amounts of three genes transcript normalized to the *GAPDH* gene. The x-axis indicates sampling times. Susceptible wheat Chinese Spring infiltration with GST protein was standardized as 1. Values are means ± SEM of three independent biological replicates. Significant differences were assessed using one-way analysis of variance (ANOVA) and Duncan’s multiple range test (DMRT) (*p < 0.05).

As the αIV helix is indispensable for TaPR1-4 interaction with TaTLP1, we speculated that the αIV helix was the functional region of TaPR1-4 that contributed to resistance to *Pt*. Truncated variants of C_Δ__128–164_-TaPR1-4 (+αIV) and C_Δ__119–164_-TaPR1-4 (-αIV) were ligated to the pGEX-6P-3 vector to express in *E. coli*, and all the pure proteins were observed by SDS-PAGE analysis [Figure 3A-(3–4), lane 5–6 and Supplementary Figure 5]. However, growth-inhibition assays showed that there was no significant difference in the germination of urediniospores and hyphal growth between the C_Δ__128–164_-TaPR1-4 (+αIV) and C_Δ__119–164_-TaPR1-4 (-αIV) (Figures 3E,F,J,K), indicating that the C_Δ__128–164_-TaPR1-4 (+αIV) and C_Δ__119–164_-TaPR1-4 (-αIV) peptides had the same antifungal activity, and the αIV helix did not affect the function of TaPR1-4.

**FIGURE 5:**
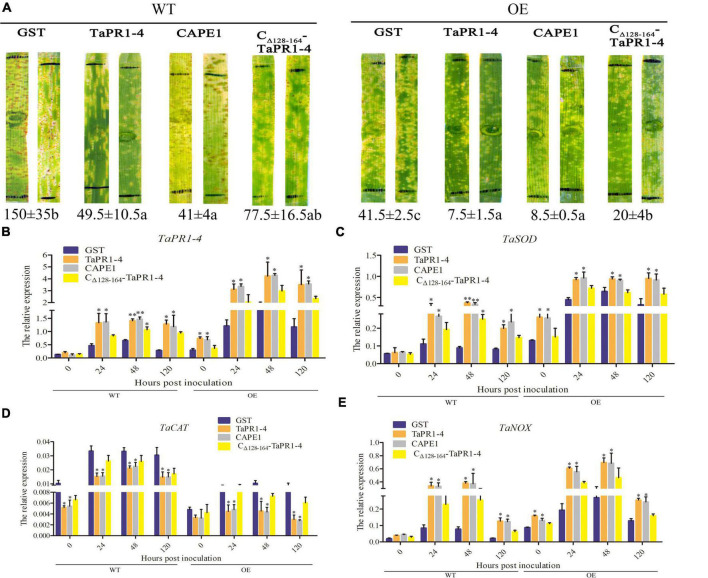
CAPE1 mediates TaTLP1-induced defense responses. **(A)** GST, TaPR1-4, CAPE1, and C_Δ__128–164_-TaPR1-4 (0.1 mg/ml) were infiltrated into the first and second leaves of TaTLP1-OE and WT lines (WT, JW1). Black lines indicate the infiltration zones. The numbers of urediniospores were quantified using Image J software. **(B–E)** Relative expression is expressed as fold change relative to mock-inoculated plants at 0 hpi. The y-axis indicates the amount of resistance-related genes transcript normalized to the *GAPDH* gene. The x-axis indicates sampling time. TaTLP1-OE and WT lines infiltration with GST protein was standardized as 1. Values are means ± SEM of three independent biological replicates. Significant differences were assessed using one-way analysis of variance (ANOVA) and Duncan’s multiple range test (DMRT) (*p < 0.05, **p < 0.01).

Sung et al. (2021) reported that the CAPE1 motif in TaPR1-1 can induce an immune response and repress infection by *Parastagonospora nodorum*. To assess the requirement of the CAPE1 region within the TaPR1-4 protein for disease repression, the CAPE1 peptide (amino acids 154–164) of TaPR1-4 was synthesized and ligated to the pGEX-6P-3 vector to express in *E. coli* [Figure 3A-(5), lane 7 and Supplementary Figure 5]. The wild-type TaPR1-4 [Figure 3A-(1), lane 3 and Supplementary Figure 5] and N-terminus truncations N_Δ__25–64_-TaPR1-4 pure protein [from our previous study, Wang F. et al. (2020)] [Figure 3A-(2), lane 4 and Supplementary Figure 5] that contain CAPE1 used as positive controls were observed by SDS-PAGE analysis. Antifungal activity results showed that the hyphae lengths treated with N_Δ__25–64_-TaPR1-4, TaPR1-4, and CAPE1 peptide were significantly shorter than the negative control groups (elution buffer, sterile water, and GST) (Figure 3), indicating that the germination of urediniospores and hyphal growth were significantly restricted with N_Δ__25–64_-TaPR1-4, TaPR1-4, and CAPE1 peptide. In addition, we found that residues A114 and V118 in TaPR1-4 and TaPR1-1 differed from other TaPR1s according to multiple sequences alignment of seven TaPR1 proteins (Supplementary Figure 4). To identify whether amino acids A114 and V118 are related to the disease resistance function of TaPR1-4, A114 and V118 in TaPR1-4 were replaced with S, respectively, and growth-inhibition assays showed no effects on disease response (Supplementary Figure 6). Taken together, all these results emphasized that the CAPE1 peptide of TaPR1-4 plays a major role in the antifungal activity, αIV helix just has a minor role in antifungal activity.

### CAPE1 Enhances Wheat Resistance to *Puccinia triticina*

To further demonstrate whether CAPE1 is indispensable for the antifungal activity of TaPR1-4 protein, the TaPR1-4 and CAPE1 proteins were infiltrated into leaves of susceptible wheat Chinese Spring, respectively. First, elution buffer, sterile water, and pGEX-6P-3 vector (GST) as negative control were infiltrated into wheat leaves to test whether contaminants from *E. coli* may elicit host immunity and prime resistance against leaf rust. After 14 days post inoculated (dpi) with *Pt* race PHNT, phenotype observation, and DAB staining results showed that GST had no effect on phenotype and H_2_O_2_ accumulation, indicating that the expression system can be used for wheat infiltration assays (Supplementary Figure 7). There were lots of urediniospores on leaves infiltrated with GST as a negative control at 14 dpi (Figure 4A). However, similar to TaPR1-4, the number of urediniospores on leaves infiltrated with the CAPE1 protein was significantly less than that infiltrated with C_Δ__128–164_-TaPR1-4 (+αIV) protein (Figure 4A). These results indicated that CAPE1 protein reduced the number of urediniospores and enhanced wheat resistance to *Pt*.

Quantitative PCR was conducted to test the expression levels of several resistance-related genes, including *TaPR1-4*, *TaTLP1*, and *TaSOD*, after protein infiltration. The results showed that all of these genes were upregulated at 24 hpi in different treatments, and the expression levels of *TaPR1-4*, *TaTLP1*, and *TaSOD* were higher after infiltrated with CAPE1 than C_Δ__128–164_-TaPR1-4 (+αIV) (Figures 4B–D), which suggests that *TaTLP1* and *TaSOD* genes were induced by the TaPR1-4, CAPE1, and C_Δ__128–164_-TaPR1-4 (+αIV) proteins. In the meanwhile, these results provide another proof that the CAPE1 plays a key role in TaPR1-4 protein-induced plant defense.

### CAPE1 Increases TaTLP1-Induced Defense Responses

To further assess whether CAPE1 could be related to TaTLP1-induced defense responses, the GST, TaPR1-4, CAPE1, and C_Δ__128–164_-TaPR1-4 (+αIV) proteins were infiltrated into TaTLP1-OE lines, with the susceptible wheat JW1 (WT) as a negative control. Phenotype after 14 days inoculated with fresh *Pt* race PHNT showed that more urediniospores appeared on WT lines infiltrated with GST protein than that infiltrated with TaPR1-4, CAPE1, and C_Δ__128–164_-TaPR1-4 (+αIV) (Figure 5A). In addition, we found that the number of urediniospores on WT lines infiltrated with TaPR1-4 and CAPE1 was significantly less than that infiltrated with C_Δ__128–164_-TaPR1-4 (+αIV) proteins (Figure 5A). In accordance with our previous reports (Cui et al., 2021), lots of necrotic spots were observed on TaTLP1-OE lines infiltrated with different proteins. Moreover, a few numbers of *Pt* urediniospores were produced around the necrotic spots on TaTLP1-OE lines infiltrated with GST and C_Δ__128–164_-TaPR1-4 (+αIV), but not on wheat leaves infiltrated with TaPR1-4 and CAPE1 (Figure 5A). All these results suggest that wheat leaves infiltrated with TaPR1-4 and CAPE1 showed significantly more resistance to PHNT compared to C_Δ__128–164_-TaPR1-4 (+αIV).

To further understand how CAPE1 participates in wheat resistance, the transcription levels of *TaPR1-4*, *TaSOD*, *TaCAT*, and *TaNOX* in the TaTLP1-OE and WT lines following infection with *Pt* were determined. qPCR results showed that *TaCAT* was significantly downregulated, while those of *TaPR1-4*, *TaSOD*, and *TaNOX* were significantly upregulated. Moreover, the expression levels of *TaPR1-4*, *TaSOD*, and *TaNOX* were higher after being infiltrated with CAPE1 and TaPR1-4 than that infiltrated with C_Δ__128–164_-TaPR1-4 (+αIV), but *TaCAT* expression was lower after infiltrated with CAPE1 and TaPR1-4 than C_Δ__128–164_-TaPR1-4 (+αIV) (Figures 5B–E). *TaSOD*, *TaCAT*, and *TaNOX* are related to ROS accumulation, which suggests that the TaPR1-4 or CAPE1 positively modulates wheat resistance to *Pt* in a ROS-dependent manner.

**FIGURE 6:**
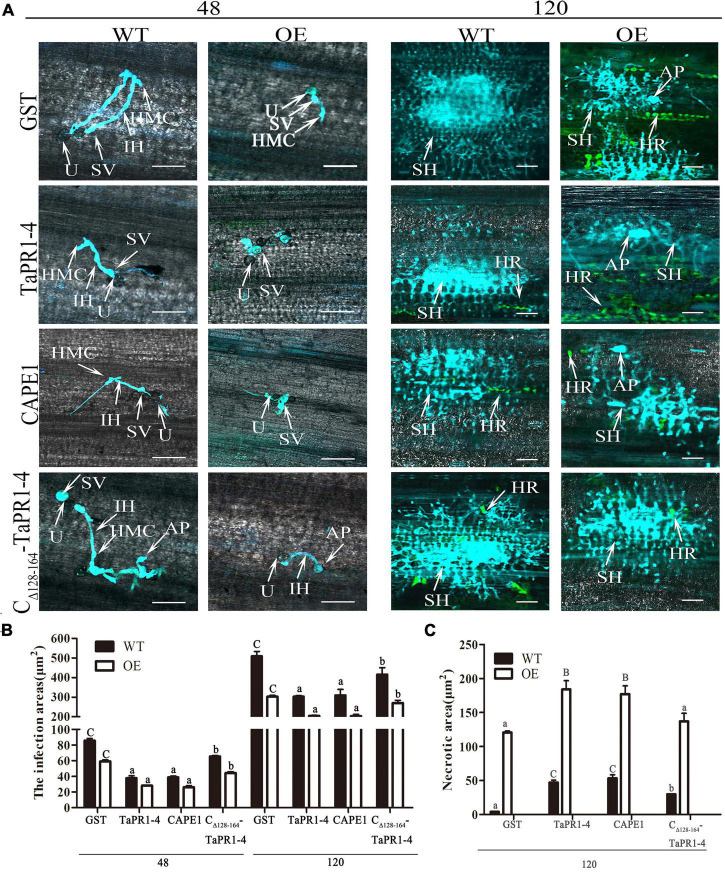
Histological changes in both WT and TaTLP1-OE lines infected with *Pt* were observed. **(A)** Histology of hyphal development and host cell death during *Pt* infection at 48 and 120 hpi. GST, TaPR1-4, CAPE1 and C_Δ__128–164_-TaPR1-4 (0.1 mg/ml) were infiltrated into first and second leaves of TaTLP1-OE and WT lines (WT, JW1). U, urediospore; AP, appressorium; IH, infection hypha; HMC, haustorial mother cell; SH, secondary hypha; SV, substomatal vesicle; HR, hypersensitive reaction (green color). Scale bars, 100 μm. **(B)** The area of infection was measured using ImageJ software. Values are means ± SEM of three independent biological replicates. Significant differences were assessed using one-way analysis of variance (ANOVA) and Duncan’s multiple range test (DMRT) with *p* < 0.05. **(C)** The necrotic area was measured using ImageJ software. A minimum of 50 infection sites were examined. Values are means ± SEM of three independent biological replicates. Significant differences were assessed using one-way analysis of variance (ANOVA) and Duncan’s multiple range test (DMRT) with *p* < 0.05.

**FIGURE 7:**
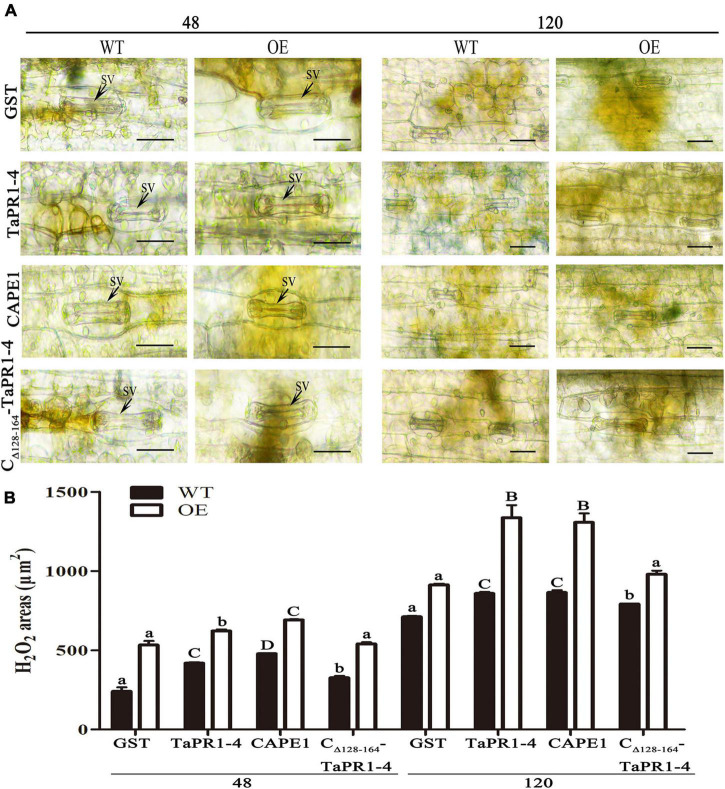
H_2_O_2_ production and necrosis were observed in these leaves at 48 and 120 hpi. **(A)** H_2_O_2_ accumulation at infection sites was detected by staining with DAB and viewed under differential interference contrast optics. GST, TaPR1-4, CAPE1 and C_Δ__128–164_-TaPR1-4 (0.1 mg/ml) were infiltrated into first and second leaves of TaTLP1-OE and WT lines. WT, JW. Scale bar, 20 μm. **(B)** ImageJ software was employed to quantify the H_2_O_2_ area. A minimum of 50 infection sites were examined. Values are means ± SEM of three independent biological replicates. Significant differences were assessed using one-way analysis of variance (ANOVA) and Duncan’s multiple range test (DMRT) with *p* < 0.05.

Histological observation of TaTLP1-OE and WT lines infected with *Pt* following CAPE1 and TaPR1-4 infiltration were recorded. It showed that the infected areas after CAPE1 and TaPR1-4 infiltration were lower than GST and C_Δ__128–164_-TaPR1-4 (+αIV) (Figures 6A,B). TaTLP1-OE lines infiltrated with CAPE1 and TaPR1-4 displayed larger necrotic areas compared to GST and C_Δ__128–164_-TaPR1-4 (+αIV) (Figure 6C), indicating that TaPR1-4 and CAPE1 in TaTLP1-OE can enhance the resistance response to *Pt* infection. In addition, we further analyzed TaPR1-4 and CAPE1-triggered ROS accumulation upon inoculation of wheat with *Pt* race PHNT. The results showed that the H_2_O_2_ accumulation was higher after infiltration with CAPE1 and TaPR1-4 than C_Δ__128–164_-TaPR1-4 (+αIV) in TaTLP1-OE and WT lines (Figure 7). Taken together, all of these results prove that CAPE1 or TaPR1-4 and TaTLP1 function additively in wheat resistance to *Pt* infection.

## Discussion

Plants have evolved defense responses against most pathogens. One mechanism of defense known as pattern-triggered immunity (PTI) involves cell-surface pattern-recognition receptors (PRRs) that mediate defense signaling (Ngou et al., 2021). Production of PR1 protein is induced by PTI. Previously, we showed that TaPR1-4 was involved in wheat defense in response to a *Pt* attack (Gao et al., 2015). RNA-seq analysis in this study showed that *TaPR1-4* is most induced by *Pt* among all *TaPR1* genes in the *Lr19* incompatible response. Then, SignalP 5.0 prediction showed that a robust signal peptide of 24 amino acids is present in TaPR1-4 protein and the cleavage site is at a position of maximum Y-score after the 24th amino acid (Supplementary Figure 8). In our previous study, co-localization techniques were used to confirm that TaTLP1 interacts with TaPR1 in the plant cell apoplast (Wang F. et al., 2020). Therefore, *TaPR1-4* was further studied to determine the underlying molecular mechanism of defense against *Pt*. We have reported that BSMV-induced *TaPR1* gene silenced wheat plants exhibited obviously compromised resistance (Wang F. et al., 2020), which suggests that *TaPR1* is involved in *Lr19*-mediated wheat defense in response to leaf rust attack.

TaTLP1–TaPR1 interaction positively modulates wheat resistance to *Pt* (Wang F. et al., 2020), which provides a basis for further investigating the mechanism that *PR* genes play in plants’ defense against pathogens. In this study, different deletion mutants were constructed according to the protein structure of TaPR1-4. We found that TaPR1-4 interacting with TaTLP1 *via* the αIV helix was involved in wheat defense response to *Pt* pathogen attack. These results confirmed an earlier report that the αIII and αIV helices of PR1 likely interacted with other proteins (Kirby et al., 2002). However, we found that αIV helix had a minor effect on the antifungal activity of TaPR1-4 *in vivo* or *in vitro*, indicating that TaPR1–TaTLP1 interaction was not required for *TaPR1* or *TaTLP1*-mediated wheat defense against *Pt*. Similarly, Sung et al. (2021) reported that SnTox3–TaPR1 interaction was not required for Snn3-dependent SnTox3-mediated necrosis, but that the CAPE1 part of TaPR1-1 was able to induce a plant immune response and repress *P. nodorum* infection. Bi et al. (2020) also reported that *Puccinia striiformis* f. sp. *tritici* secreted the effector PNPi that targeted the CAPE1 region in TaPR1a protein and suppressed the expression of *PR* genes. Until now, the antifungal activity of CAPE1 against *Pt* had been verified. Here, αIV helix plays major roles in TaPR1–TaTLP1 interaction but has minor roles in antifungal activity. Conversely, the CAPE1 motif is more important in the antifungal function and less important in interaction with TaTLP1. All this evidence suggests that TaPR1-4 has both TaTLP1 dependent and independent functions. As CAPE1 could be cleaved from TaPR1 (Sung et al., 2021), we suspect that CAPE1 may function separately after release from TaPR1-4 while the remaining N-terminal forms complex with TaTLP1 to play a disease resistance function.

Our previous studies have proved that TaTLP1-OE had significant levels of resistance to common root rot and leaf rust, indicating that *TaTLP1* had the potential to be deployed to defense both pathogens in the field (Cui et al., 2021). Moreover, we identified that TaTLP1 interacts with TaPR1 to contribute to wheat defense responses to leaf rust fungus (Wang F. et al., 2020). In this study, we found that TaTLP1 and TaPR1-4 or CAPE1 function additively. In addition, CAPE1 in TaPR1-4 activated the transcription level of the resistance-related genes regulating ROS accumulation such as *TaNOX*, *TaSOD*, and *TaCAT*. As we all know, a high concentration of ROS induces cell death in plants (Dimitrov and Frank, 2012). Among them, *TaSOD* catalyzes the conversion of superoxide anion to O_2_ and H_2_O_2_ (Fukai and Ushio-Fukai, 2011), and *TaNOX* is known to generate H_2_O_2_ (Lambeth, 2004). *TaCAT*, which is the major ROS-scavenging enzymes, could eliminate ROS accumulation (Mittler et al., 2004). Although it confirmed that CAPE1 enhances TaTLP1 defense response to *Pt via* ROS, in the future, it is necessary to demonstrate whether or how TaPR1-4 or CAPE1 plays a role in the TaTLP1 pathway.

Although the function of PR1 protein has remained elusive for decades, induction of host defense signaling through peptide release from precursors has been previously reported (Zhang et al., 2020). For example, TaPR1-enhanced resistance to infection by *P. nodorum* in wheat was dependent on the release of the TaCAPE1 peptide embedded within TaPR1 by an unidentified serine protease (Sung et al., 2021). CAPE1 of TaPR1 peptide consists of 15 amino acids (CNYxPxGNxxxxxPY-), including the CNYx of CAPE1, which is required for cleavage from TaPR1 (Chen et al., 2014). The identical CNYx sequence of TaPR1-1 is present in TaPR1-4 (Supplementary Figure 4). Whether the cleavage mechanism of CAPE1 in TaPR1-1 is the same in TaPR1-4 needs to be confirmed.

Salicylic acid (SA) is elevated in response to pathogen challenges (Islam et al., 2020). NPR1 converts to a monomeric state and translocates defense signaling to the nucleus (Tada et al., 2008; Wang X. et al., 2020). NPR1 interacts with TGA, and WRKY interacts with TGA2 and TGA5 in the nucleus. SA signal transduction activates the expression of a battery of *PR* genes, such as *TaPR1* and *TaTLP1* (Hussain et al., 2018; Wang F. et al., 2020). We propose a model that TaTLP1 binds to TaPR1-4 *via* the αIV helix and plays a broad role in basal plant immunity *via* the activity of its C-terminal CAPE1 peptide to regulate ROS generation (Supplementary Figure 9). To the best of our knowledge, this is the first direct evidence demonstrating that CAPE1 of TaPR1-4 can affect infection by *Pt*. These results provide a foundation for finally understanding the function of TaPR1 and the role it plays in wheat–*Pt* interaction.

## Data Availability Statement

The datasets presented in this study can be found in online repositories. The names of the repository/repositories and accession number(s) can be found in the article/Supplementary Material.

## Author Contributions

FW and HW conceived the research plans and wrote and revised the manuscript. FW, SS, ZC, and WW performed most of the experiments. SS, ZC, and LM analyzed the data. FW and CZ generated the pictures. FW and SS contributed reagents and materials and to interpretation of the results. HW and DL supervised this experiment. All authors contributed to the article and approved the submitted version.

## Conflict of Interest

The authors declare that the research was conducted in the absence of any commercial or financial relationships that could be construed as a potential conflict of interest.

## Publisher’s Note

All claims expressed in this article are solely those of the authors and do not necessarily represent those of their affiliated organizations, or those of the publisher, the editors and the reviewers. Any product that may be evaluated in this article, or claim that may be made by its manufacturer, is not guaranteed or endorsed by the publisher.

## Footnotes

1
https://web.expasy.org/protparam/

2
https://plants.ensembl.org/Triticum_aestivum/Info/Index

3
https://services.healthtech.dtu.dk/service.php?SignalP-5.0
