# Supplementary material for: TaPR1 Interacts With TaTLP1 via the αIV Helix to Be Involved in Wheat Defense to Puccinia triticina Through the CAPE1 Motif
Source: Front Plant Sci. 2022 May 26;13:874654. doi: 10.3389/fpls.2022.874654 (PMC9199852; doi:10.3389/fpls.2022.874654)
Supplement: Supplementary file 1 [file Table_1.DOCX]

Supplementary Table 1 Primers used in this study

| Primer name | Primer sequence |
| --- | --- |
| Q-PR1-1-F | TGCACGTTCCTATCTACTG |
| Q-PR1-1-R | CAAGGTACCGTAAAAATGCACA |
| Q-PR1-7-F | ATATGCATCATGCATGCATGCGA |
| Q-PR1-7-R | ATTACCTGAATCATAACGGTACA |
| Q-PR1-19-F | CTAGGCTATGCATGCGTGCGT |
| Q-PR1-19-R | AGCCTGCTAAAGTGTCACAA |
| Q-PR1-9-F | GCAGCGCGTATATTGCATAAAGAAT |
| Q-PR1-9-R | CTTTATTTTCATTCATAAGCACTGC |
| Q-PR1-16-F | AACTACGAGGGGGTGAGCCCATACT |
| Q-PR1-16-R | TCGTAACACATTATCCCATTAGCCG |
| Q-PR1-20-F | GTCTTCATCACCTGCAACTACAATC |
| Q-PR1-20-R | TGTAGACGTATAAATATTCAAACCA |
| q-GAPDH-F | TGCCTTGCTCGTCTTGCTAA |
| q-GAPDH-R | CTTGATGGAAGGACCATCAAC |
| qTaPR1-4-F | CAATAACCTCGGCGTCTTCATC |
| qTaPR1-4-R | ATTTACTCGCTCGGTCCCTC |
| qTaTLP1-F  qTaTLP1-R | GGGATCCATGGCGACCTCCGCGGTGCTC  CCAAGCTTTCATGGACAGAAGGTGATCTGGTC |
| q-TaSOD-F | GGCTCTCCAAGGTCGTGT |
| q-TaSOD-R | GGGTTGCCGTTGTTGTAG |
| q-TaCAT-F | GCCTGTGTTTTTTATCCGAGA |
| q-TaCAT-R | CAGGTGCCTCCAACAGTAACA |
| q-TaNOX-F | AGATGGAGGAAGAGGAGGATA |
| q-TaNOX-R | CGTAAACGCTTGTGAGGTAGT |
| TaTLP1 prey-F | GAATTC GCCACCTTCTACATCAAGAACAACTGC |
| TaTLP1 prey-R | GGATCC TCATGGACAGAAGGTGATCTGGTAG |
| TaPR1 Biat-F | GAATTC CAGAACTCGCCTCAGGACTACCTCTCAC |
| TaPR1 Biat-R | GGATCC GTATGGTTTCTGTCCAATGATATTCCCG |
| N_Δ25-64_-TaPR1-4-F | GAATTC CCCAGGCGCAGAACTCGCCTCA |
| N_Δ25-64_-TaPR1-4-R | GGATCC GTATGGTTTCTGTCCAATGATATTCCCG |
| C_Δ119-164_-TaPR1-4-F | GAATTC CAGAACTCGCCTCAGGACTA |
| C_Δ119-164_-TaPR1-4-R | GGATCC CACCTTCCCCGCCGCGCA |
| C_Δ113-164_-TaPR1-4-R | GGATCC GCAGGTGTTGGAGCCGTAG |
| C_Δ128-164_-TaPR1-4-R | GGATCC GCCACACCACCTGCGTGTAGTGC |
| C_Δ143-164_-TaPR1-4-R | GGATCC GTTATTGTTGCAGACGACGCGGG |
