# Supplementary material for: TaPR1 Interacts With TaTLP1 via the αIV Helix to Be Involved in Wheat Defense to Puccinia triticina Through the CAPE1 Motif
Source: Front Plant Sci. 2022 May 26;13:874654. doi: 10.3389/fpls.2022.874654 (PMC9199852; doi:10.3389/fpls.2022.874654)
Supplement: Supplementary file 2 [file Table_2.DOCX]

Supplementary Table 2 *TaPR1* genes identified in the RNA-seq library

| Protein name | *Triticum aestivum* (IWGSC) | Accession number | Size(bp) | Protein length | SP | Molecular weight^a^ | p*I* | Group | Chr^b^ | Location^b^ |
| --- | --- | --- | --- | --- | --- | --- | --- | --- | --- | --- |
| TaPR1-1 | TraesCS5B02G181500 | HQ541961 | 495 | 164 | 24 | 17634.89 | 8.7 | I | 5B | Extracellular |
| TaPR1-4 | TraesCS7D02G161200 | HQ541964 | 495 | 164 | 24 | 17536.74 | 8.6 | I | 7D | Extracellular |
| TaPR1-7 | TraesCS5B02G443400 | HQ541967 | 498 | 165 | 22 | 17774.45 | 4.2 | III | 5B | Extracellular |
| TaPR1-9 | TraesCS5A02G439700 | HQ541969 | 501 | 166 | 23 | 17831.49 | 4.3 | III | 5A | Extracellular |
| TaPR1-16 | TraesCS5D02G446900 | HQ541976 | 504 | 167 | 24 | 17818.64 | 4.5 | III | 5D | Extracellular |
| TaPR1-19 | TraesCS5A02G439800 | HQ541979 | 504 | 167 | 24 | 17801.64 | 4.8 | III | 5A | Extracellular |
| TaPR1-20 | TraesCS7D02G201400 | HQ541980 | 522 | 173 | 25 | 18800.04 | 8.3 | II | 7D | Extracellular |

a: Prediction of protein molecular weight by online software (<https://web.expasy.org/protparam/>).

b: The chromosomal localization and subcellular localization of TaPR1 proteins were determined by online software EnsemblPlants.
