# Supplementary material for: TaPR1 Interacts With TaTLP1 via the αIV Helix to Be Involved in Wheat Defense to Puccinia triticina Through the CAPE1 Motif
Source: Front Plant Sci. 2022 May 26;13:874654. doi: 10.3389/fpls.2022.874654 (PMC9199852; doi:10.3389/fpls.2022.874654)
Supplement: Supplementary file 17 [file Data_Sheet_15.docx]

Supplementary Material


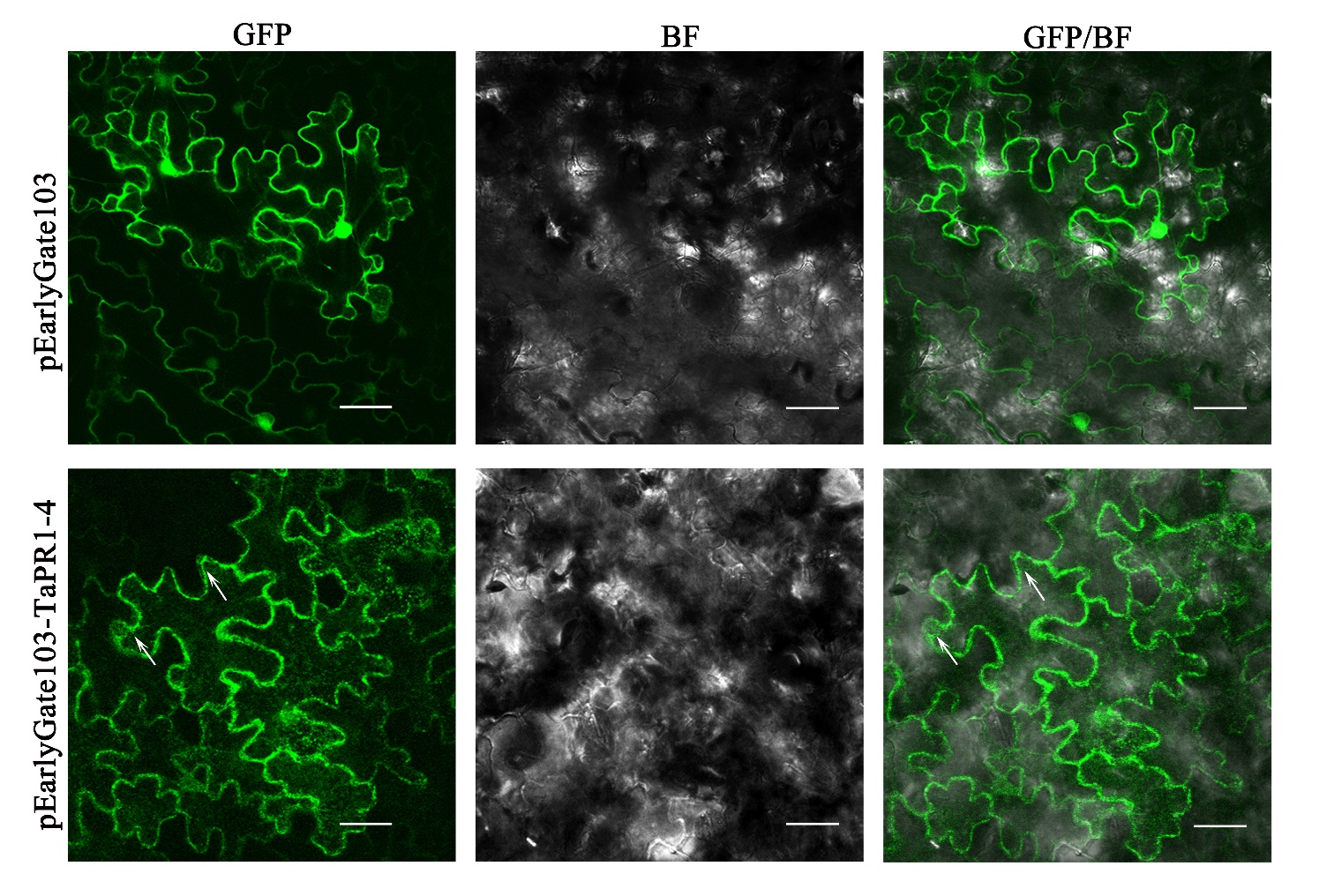
Supplementary Figure 1

Supplementary Figure 1 TaPR1-4 is secreted and localizes in the apoplastic space of *N. benthamiana* epidermal cells. The proteins were expressed in *N. benthamiana* following agroinfiltration. Fluorescence was detected in epidermal cells of the infiltrated leaves by confocal microscopic 48 h after agroinfiltration. The white arrow showing TaPR1-4 was visualized in the apoplastic space (AP) as evidenced by the plasmolysis. Scale bars, 50 mm.

Supplementary Figure 2


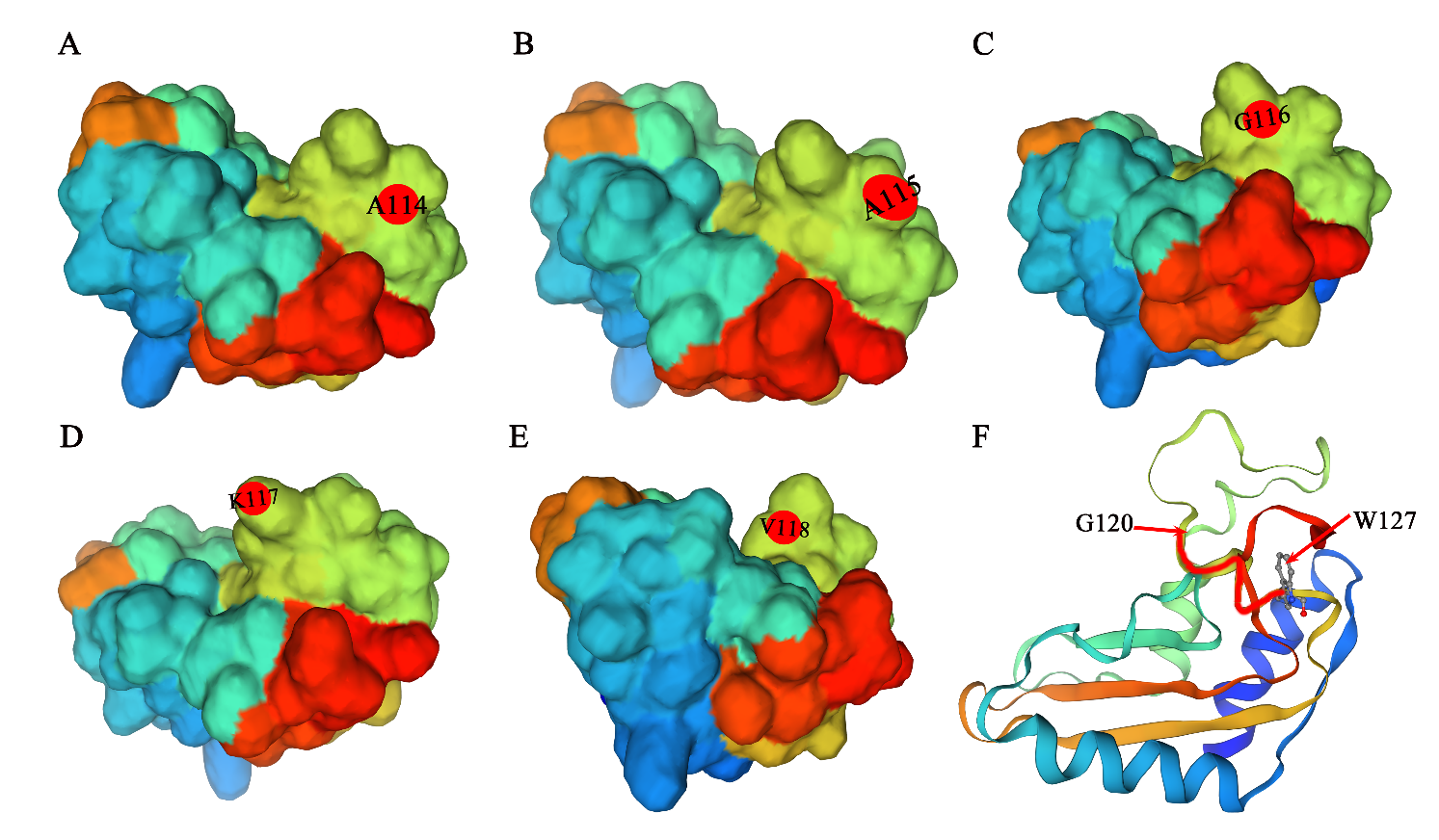


Supplementary Figure 2 The senior structure of TaPR1-4 protein. Amino acids A114, A115, G116, K117, V118 of TaPR1-4 were shown as a-e. Cartoon representation amino acids from G120 to W127 were αⅣ helix; it was indicated by red curve. The arrow marks the position from G120 to W127 was shown as f. Protein structure was made using software SWISS-MODEL.

Supplementary Figure 3


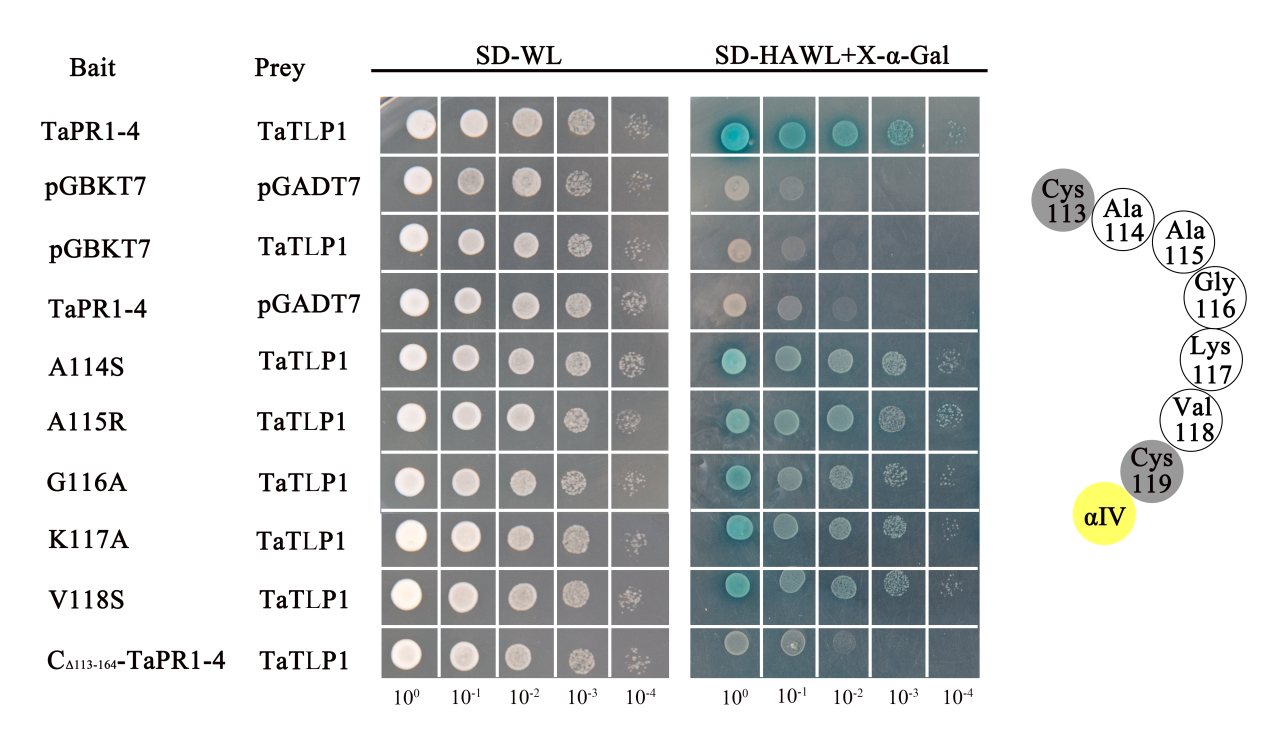


Supplementary Figure 3 Site-specific mutagenesis from A114 to V118 of TaPR1-4 weakened its interaction with TaTLP1. Site-specific mutation of TaPR1-4 including A114 replace with Ser (A114S), A115 replace with Arg (A115R), G116 replace with Ala (G116A), K117 replace with Ala (K117A), V118 replace with Ser (V118S) as bait, TaTLP1 as prey. Each of the site-specific mutation was co-transformed with TaTLP1 into yeast. Positive control is TaPR1-4-TaTLP1. TaPR1-4 and pGADT7, TaTLP1 and pGBKT7, pGADT7 and pGBKT7 were used as the negative controls. All transformants are able to grow on synthetic dropout medium without leucine and tryptophan (SD-WL) medium. Yeast colonies that were able to grow on selective medium (SD medium without leucine, tryptophan, histidine, and adenine supplemented with X-α-Gal and AureobasidinA [SD-HAWL]) and displayed blue coloration confirmed the protein-protein interaction.

Supplementary Figure 4

αⅠ

βA

αⅡ

βB

αⅢ

3_10_

αⅣ

βC

βD

Supplementary Figure 4 Protein sequences alignment about seven TaPR1 proteins. Sequences come from NCBI (https://www.ncbi.nlm.nih.gov/). Gene ID: *TaPR1-4* from our previous study, ID: HQ848391, *TaPR1-1*’s gene ID: HQ541961, *TaPR1-7*’s gene ID: HQ541967, *TaPR1-9*’s gene ID: HQ541969, *TaPR1-16*’s gene ID: HQ541976, *TaPR1-19*’s gene ID: HQ541979, *TaPR1-20*’s gene ID: HQ541980. The position selected in the blue box is 113-127 aa, the black arrow point A114 and V118, the red box is CAPE1.

Supplementary Figure 5


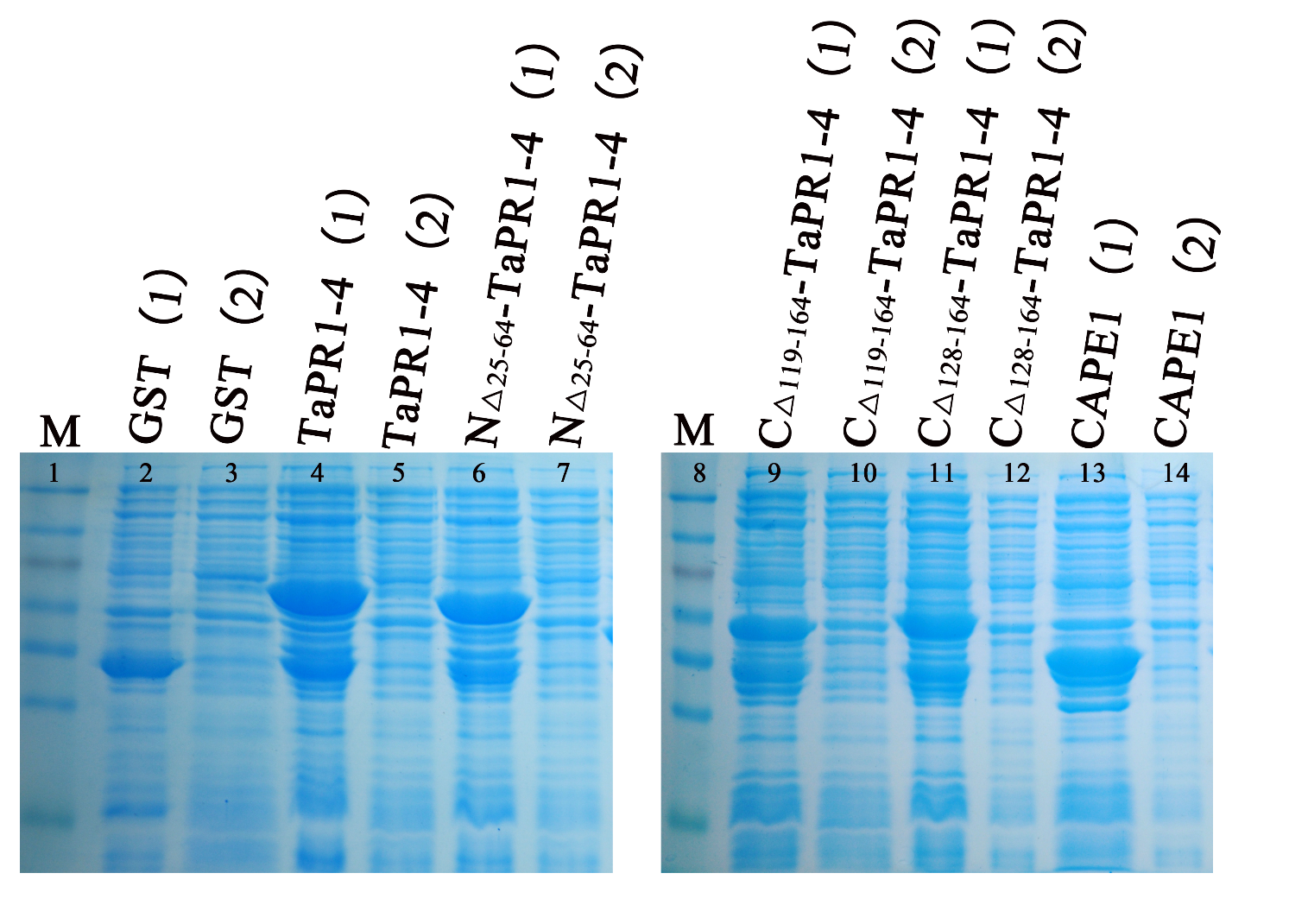


Supplementary Figure 5 The protein electrophoretogram of TaPR1-4 and four mutants induced by IPTG. (1) The crude protein induced by IPTG. (2) The crude protein without IPTG. Truncated TaPR1-4 constructs were generated including N-terminal deletion of residues 25 to 64 (N_△25-64_-TaPR1-4), and C-terminal deletion of residues 119 to 164 (C_△119-164_-TaPR1-4), deletion of residues 128 to 164 (C_△128-164_-TaPR1-4) and deletion of residues 25 to 153 (CAPE1).

Supplementary Figure 6


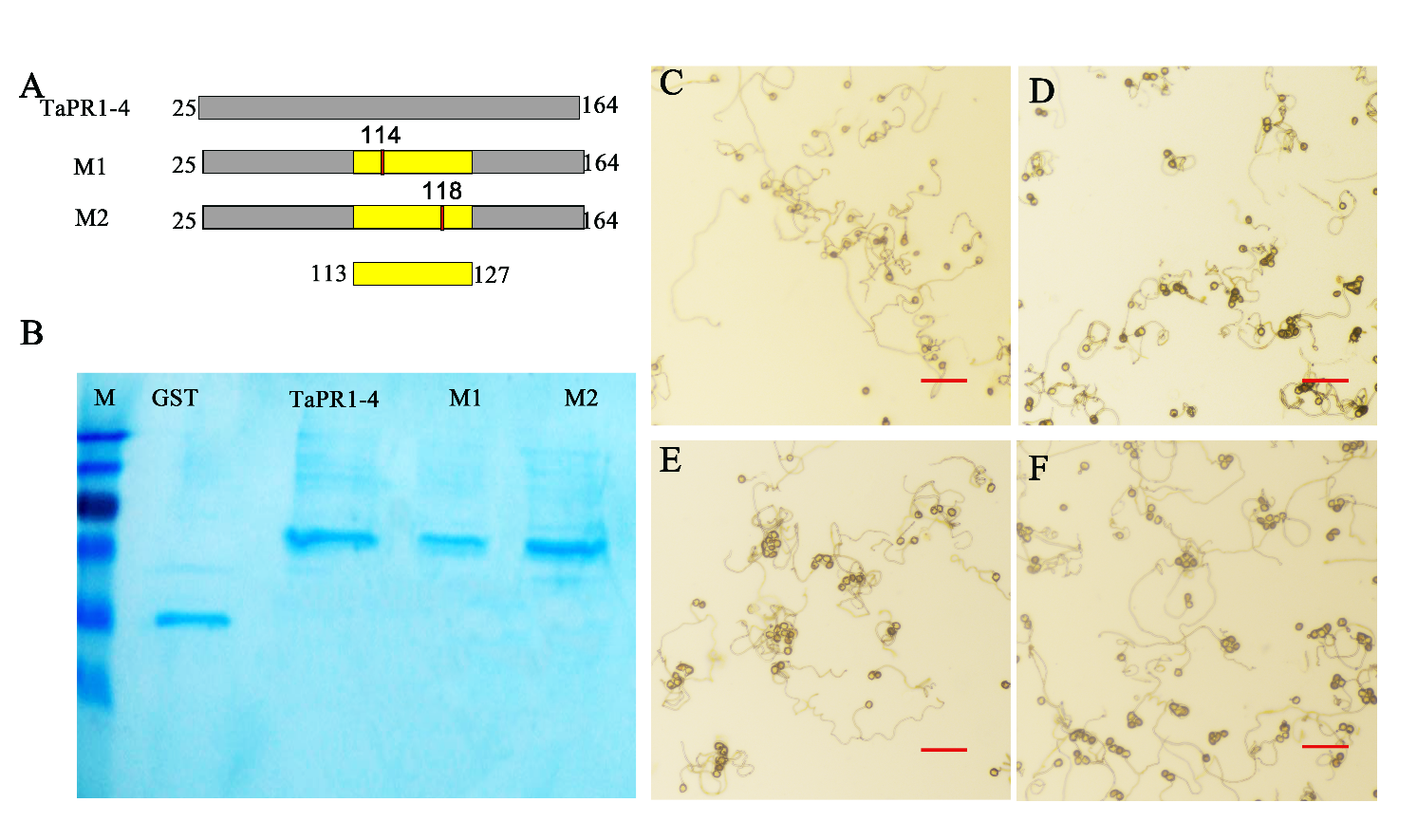


Supplementary Figure 6 The antifungal activity of mutants M1 and M2 *in vitro*. (A) Schematic of constructing site-directed mutagenesis based on protein secondary structure. (B) Using SDS-PAGE electrophoresis detected purified proteins. Lane 1 to lanes 5 show Marker, GST, TaPR1-4, M1 (replace A114 with S) and M2 (replace V118 with S), these pure protein were expressed by *E.coli*. The proteins were stained with Coomassie Brilliant Blue. (C-F) The presence of pure protein inoculation on the 2% agar medium under 25˚C dark for 12 hours, c to f show GST, TaPR1-4, M1 and M2. Photographs were taken at 10× magnification. Scale bar, 100 μm.

Supplementary Figure 7


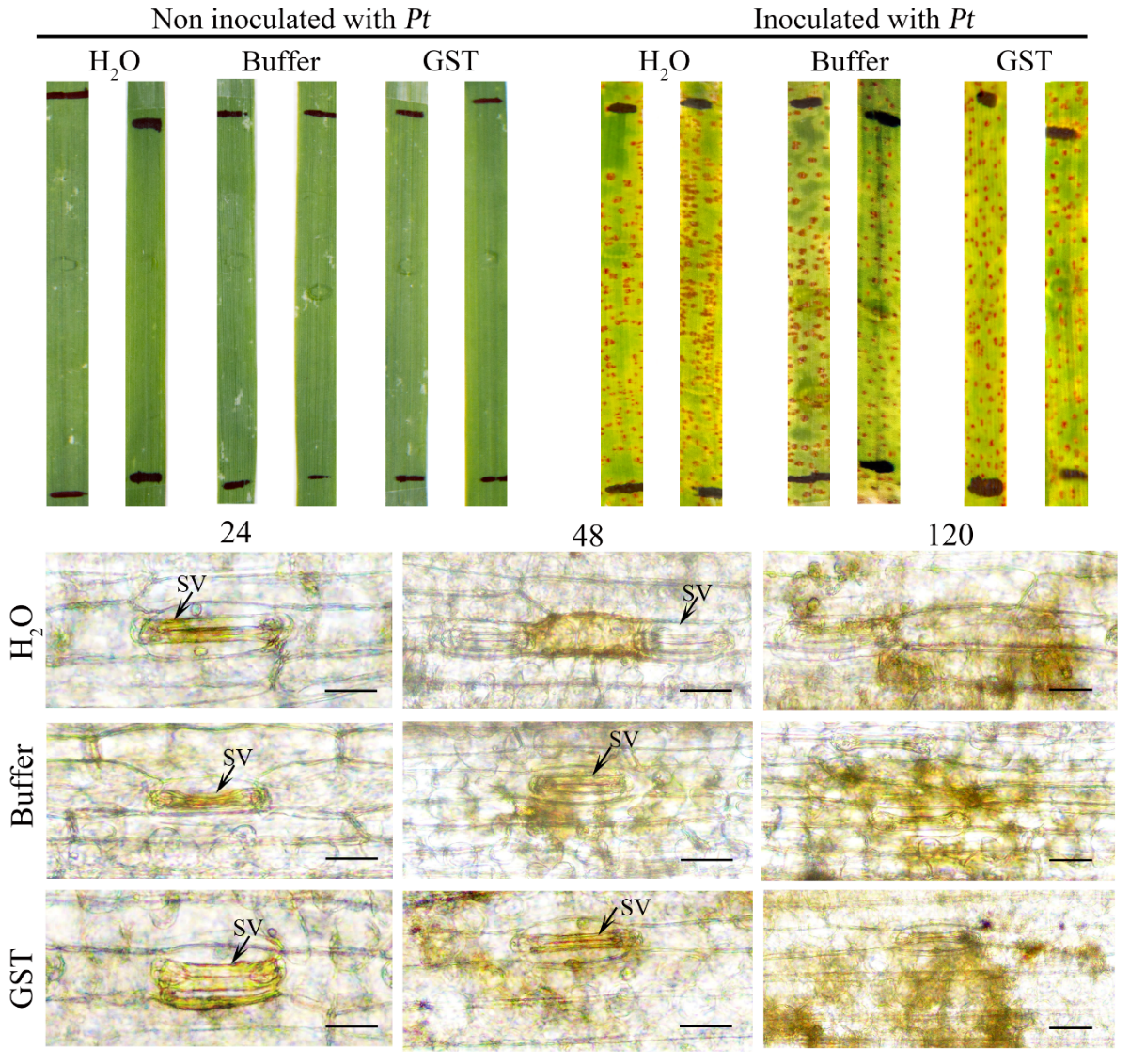


**Supplementary Figure 7 Phenotypes and H_2_O_2_ accumulation were observed after infiltration buffer and GST in wheat leaves. (A)** H_2_O, buffer and GST were infiltrated into first leaves of Chinese Spring. Black lines indicate the infiltration zones. The phenotype was observed after 14 days. **(B)** H_2_O_2_ accumulation at infection sites was detected by staining with DAB and viewed under differential interference contrast optics. Scale bar, 20 μm.

Supplementary Figure 8


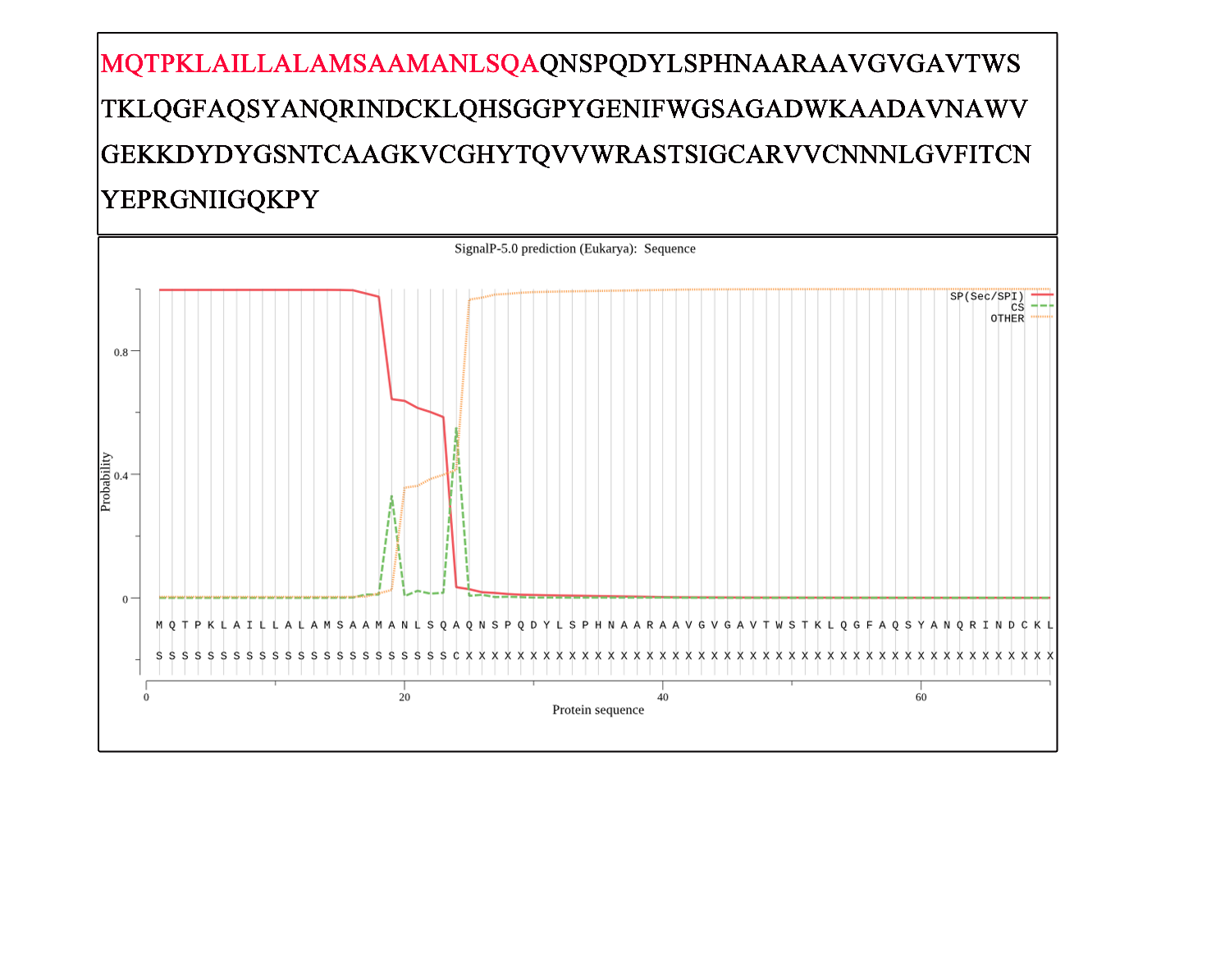


**Supplemental Figure 8 Characteristics of the translated protein of TaPR1-4.** Predicted signal peptide sequence (highlighted with red font) of the TaPR1-4 protein. The graphical output of SignalP 5.0 predictions.

Supplementary Figure 9


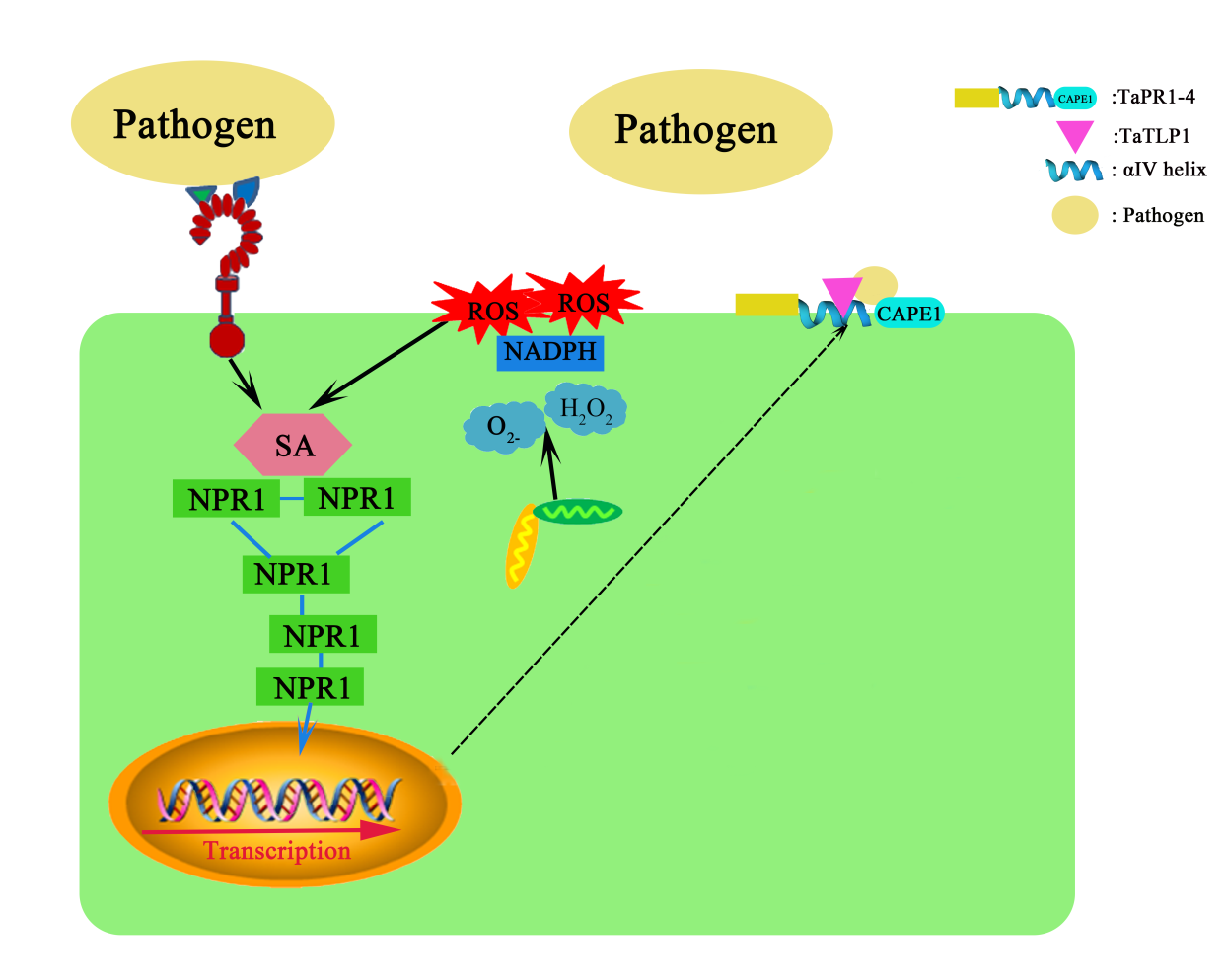


**Supplementary Figure 9 CAPE1 of TaPR1-4 positively contributes to wheat resistance to *Pt*.** Upon pathogen challenge, the plant defense signaling molecule SA increases, NPR1 converts to a monomeric state translocate defense signaling to nucleus, *WRKY*, *NAC* and *TGA-bZIP* transcriptional factors can activate the expression of a battery of *PR* genes. The TaTLP1 could bind TaPR1-4 via αⅣ helix. So TaPR1-TaTLP1 and CAPE1 positively contributes to wheat resistance to *Pt*, and regulates ROS generation in the interaction between wheat and *Pt.*
